# Species-informative SNP markers for characterising freshwater prawns of genus *Macrobrachium* in Cameroon

**DOI:** 10.1371/journal.pone.0263540

**Published:** 2022-10-03

**Authors:** Judith G. Makombu, Evans K. Cheruiyot, Francesca Stomeo, David N. Thuo, Pius M. Oben, Benedicta O. Oben, Paul Zango, Eric Mialhe, Jules R. Ngueguim, Fidalis D. N. Mujibi

**Affiliations:** 1 Department of Fisheries and Aquatic Resources Management, Faculty of Agriculture and Veterinary Medicine, University of Buea, Buea, Cameroon; 2 USOMI Limited, Nairobi, Kenya; 3 Biosciences Eastern and Central Africa—International Livestock Research Institute (BecA-ILRI) Hub, Nairobi, Kenya; 4 Australian National Wildlife Collection, National Research Collections Australia, CSIRO, Canberra, Australia; 5 Institute of Fisheries and Aquatic Sciences, University of Douala, Yabassi, Cameroon; 6 Concepto Azul, Cdlavernaza Norte, Guayaquil, Ecuador; 7 Institute of Agriculture Research for Development (IRAD), Kribi, Cameroon; Shanghai Ocean University, CHINA

## Abstract

Single Nucleotide Polymorphisms (**SNPs**) are now popular for a myriad of applications in animal and plant species including, ancestry assignment, conservation genetics, breeding, and traceability of animal products. The objective of this study was to develop a customized cost-effective SNP panel for genetic characterisation of *Macrobrachium* species in Cameroon. The SNPs identified in a previous characterization study were screened as viable candidates for the reduced panel. Starting from a full set of 1,814 SNPs, a total of 72 core SNPs were chosen using conventional approaches: allele frequency differentials, minor allele frequency profiles, and Wright’s Fst statistics. The discriminatory power of reduced set of informative SNPs were then tested using the admixture analysis, principal component analysis, and discriminant analysis of principal components. The panel of prioritised SNP markers (i.e., N = 72 SNPs) distinguished *Macrobrachium* species with 100% accuracy. However, large sample size is needed to identify more informative SNPs for discriminating genetically closely related species, including *M*. *macrobrachion* versus *M*. *vollenhovenii* and *M*. *sollaudii* versus *M*. *dux*. Overall, the findings in this study show that we can accurately characterise *Macrobrachium* using a small set of core SNPs which could be useful for this economically important species in Cameroon. Given the results obtained in this study, a larger independent validation sample set will be needed to confirm the discriminative capacity of this SNP panel for wider commercial and research applications.

## Introduction

Freshwater prawns of the genus *Macrobrachium* Bate, 1868 (Crustacea, Decapoda, Palaemonidae) are a highly diverse group of decapod crustaceans of high economic importance globally. They occur in diverse habitats worldwide, from brackish estuarine to upland streams of the tropics and subtropics [1, 2]. A total of 240 *Macrobrachium* species are presently known [3–5], although it is difficult to estimate the correct species richness of *Macrobrachium*, as new taxa are often described every year. For example, *M*. *ayeyarwadiense* [5] and *M*. *chainatense* [6] were recently described by researchers in Myanmar and Thailand, respectively.

Ecologically, *Macrobrachium* plays a critical role in stream food webs because it serves as an intermediate consumer, linking the production of periphyton and detritus with higher trophic groups [7]. Economically, *Macrobrachium* serves as important food resources for carnivorous fish and humans, it is amongst the main target species for fisheries and aquaculture [8] and it sustains most viable artisanal and commercial fisheries in the West Africa Sub-region [9]. However, the *Macrobrachium* fauna of West Africa is poorly understood.

The main taxonomic records date back to the general investigations of decapod crustaceans in West Africa [10, 11], which reported 10 species of *Macrobrachium* in this region including four in Cameroon. Recent studies pointed out the higher species richness of Cameroonian *Macrobrachium* [12–14] and increased the number of known species from four to six: *M*. *vollenhovenii*, *M*. *macrobrachion*, *M*. *chevalieri*, *M*. *sollaudii*, *M*. *dux*, *M*. *felicinum*. All these studies used morphological keys. It is well-known that morphological identification of species of this genus is quite difficult because many features used for identification are common to all known species [15]. These studies illustrate that traditional morphological characters alone are insufficient in the accurate diagnosis of the genus *Macrobrachium* for breeding and conservation purposes. Currently, farmers in Cameroon typically collect *Macrobrachium* seed (juveniles) from wild capture to rear in earthen ponds. However, given the morphological similarity of juveniles for this species, it is difficult to distinguish the other species from *M*. *vollenhovenii*, which is of high aquaculture potential. The species being studied here are of very high economic importance to Cameroonian aquaculture and as such, breeding trials are currently being conducted on *M*. *vollenhovenii* at the University of Buea, Cameroon to facilitate distribution of seed to farmers for grow-out operations.

Molecular data has proven very useful to elucidate the taxonomic relationships in morphologically variable groups of freshwater prawns [16]. Several studies have used mitochondrial DNA sequence data from the 16S rRNA and cytochrome c oxidase subunit 1 (CO1) genes to characterize Asian *Macrobrachium* taxonomy, biogeography, evolution, and life history (e.g., [15–17]). Microsatellite markers have also been developed for *M*. *rosenbergii* [18]. Overall, using molecular tools can help to accurately distinguish *Macrobrachium* species for the benefit of aquaculture farmers and for conservation purposes.

In our recent work [19], we used Diversity Arrays Technology (DArT) [20] to genotype and characterize *Macrobrachium* species from the coastal area of Cameroon using 1,814 SNPs. In that study, we identified at least four species of *Macrobrachium* based on the ADMIXTURE analysis and five species when using principal component analysis (PCA), using 1814 SNP markers and for 93 individuals from different species initially differentiated using morphological keys. In this study, we set out to identify a smaller set of informative SNP markers that can be used to characterize *Macrobrachium* species, with the aim of ultimately reducing the cost of genotyping to allow a larger number of individuals to be evaluated in future studies. This is in line with similar studies in humans [21], wildlife [22], livestock [23, 24], and crops [25]. [21] screened 432 SNPs and chose 40 informative SNP markers for forensics and paternity testing in humans. Similarly [22], screened a total of 158 SNPs and identified a suite of 35 SNPs for genetic inference of domestic cats and European wildcats, while [23] identified 48 and 96 SNPs from a set of 50K SNPs for breed assignment in cattle. Besides the cost, the informative set of markers for *Macrobrachium* species could be useful for routine use in species ancestry assignment, conservation, forensics, and breeding purposes.

Several methods have been proposed for identification of informative genetic markers for inference of population structure, such as the Delta method, which estimates allele frequency difference between pairs of populations [26], Fst variants [27], informativeness for assignment (*I*_n_) [28], and PCA [29]. These methods are closely related and gives comparable results [30]. More recently [24], used a machine learning approach (Random Forest) to select 96 informative SNPs from the 60K porcine array for use in discriminating pig breeds.

In this study, we chose a smaller set of informative SNPs for genetic characterisation of *Macrobrachium* species from a full set of 1,814 SNPs using conventional approaches: a) SNPs with high Weir & Cockerham Fst values [27], and b) SNPs defined as ‘private’ or unique for each study species because they are segregating (i.e., not fixed) in only one out of the seven populations studied. Notably, the number of populations used in this study was informed by our previous work [19].

## Materials and methods

### Samples, genotyping and quality checks

**Fig 1:**
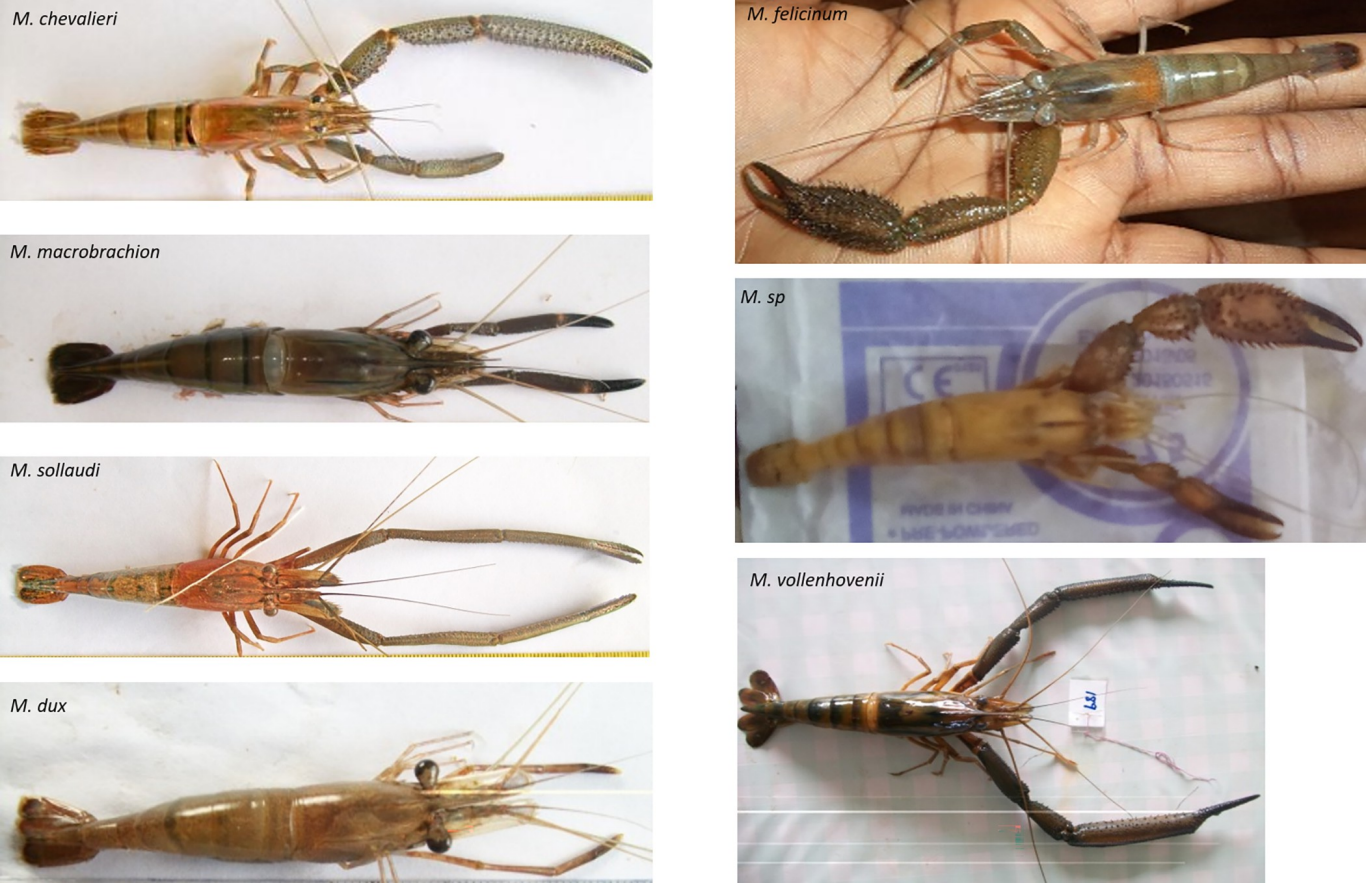
Pictures of seven *Macrobrachium* species in the coastal area of Cameroon identified based on morphological analysis using [10, 31] keys.

The dataset used in this study is part of our previous work and has been described in more detail by [19], including the sampling locations (map), morphological characterization, and genotyping. Briefly, we collected a total of 1,566 *Macrobrachium* specimens from fishermen catches between May 2015 and April 2016 covering major riverine areas and sources of these species in Cameroon: Lokoundje, Kienke, and Lobe Rivers, in the South region; at Batoke, Mabeta and Yoke rivers in the South-West region and Nkam and Wouri rivers in the Littoral region of Cameroon (comprehensive descriptions of the specimens were given by [19]). Out of these samples, a small set of 93 individuals was selected for genotyping representing seven species: 18 samples from *M*. *dux*; 18 *M*. *macrobrachion*; 18 *M*. *sollaudii*; 17 *M*. *vollenhovenii*; 12 *M*. *chevalieri*; 5 *M*. *felicinum*, and 5 *M*. sp (an undescribed species). These species were identified based on the morphological key described by [10, 31]. The images of the study species are shown in Fig 1. The new undescribed species was found to be morphologically close to *M*. *felicinum* [19] using morphological keys. However, molecular analysis showed that the individuals of this group have a very distinct genetic signature and were labelled as an undescribed species separate from the *M*. *felicinum*.

Following DNA extraction and genotyping using DArT markers [20], a total of 1,814 out of 52,834 SNPs were retained for data analysis, as described in [19]. This remaining set of markers (N = 1,814) passed quality checks based on call rate > 80% and minor allele frequency (MAF > 5%). To identify chromosomal positions, the allele sequences of these SNPs were mapped to the reference genome of *M*. *nipponense* using Basic Local Alignment Search Tool (BLAST) [32]. We used this set of markers in this study as a benchmark to test species assignment to the respective population versus the reduced set of ‘informative SNPs’.

### Identifying informative SNP markers

We used the following steps to identify ‘private’ SNPs (i.e., those segregating in only one species) for *Macrobrachium* species [*M*. *dux*, *M*. *macrobrachion*, *M*. *sollaudii*, *M*. *vollenhovenii*, *M*. *chevalieri*; *M*. *felicinum*, *M*. sp]:

1. Compute allele frequencies for each population and SNP (N = 1,814) using the Hierfstat package [33] in R [34].
2. Select SNPs that are segregating in only one population (i.e., fixed allele frequencies in six out of seven species or populations studied).
3. For the SNPs in set 2 above, select SNPs segregating with a minimum threshold of 0.03 for the alternate allele to avoid fixed SNPs.
4. Repeat the above steps (i.e., 1 to 3) for 100 runs by randomly selecting 80% of the individuals in each species for each repeat run.
5. For private SNPs in step 4, select two sets of SNPs: a) informative or ‘private SNPs’ identified in > 50% (i.e., > 50 runs) of the 100 repeated runs, and b) informative or ‘private SNPs’ identified in > 80 runs (considered as most stable core SNPs). These SNP sets will be called ‘private SNPs 50’ and ‘private SNPs 80’ panels.

**Fig 2:**
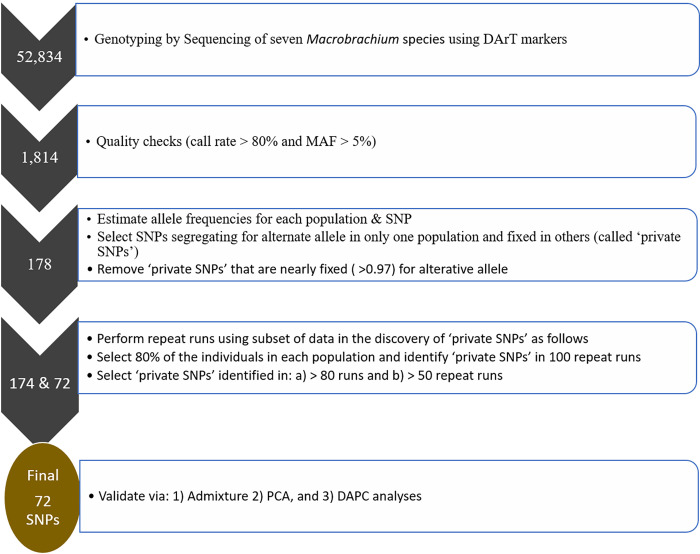
An overview of the identification and validation of ’private SNPs’ or informative SNPs. Step 1: a total of 52,834 SNPs were generated from genotyping by sequencing of Macrobrachium species. Step 2: the SNPs from ‘Step 1’ were screened for quality parameters leaving a total of 1,814 SNPs for further analysis. Step 3: the SNPs from ‘Step 2’ were used to prioritise 178 informative SNPs based on allele frequency estimates. Step 4: a total of 72 high-quality SNPs (‘private SNPs’) were selected from the 178 SNPs in ‘Step 3’ based on repeat resampling approach. Step 5: the SNPs from ‘Step 4’ were “validated” using three methods: a) PCA–principal component analysis; b) Admixture, and c) DAPC–discriminant analysis of principal components. MAF–minor allele frequency.

As an alternative approach, we computed Weir & Cockerham Fst values [27] for a full set of SNPs (N = 1,814) using PLINK software v1.9 [35]. We then selected SNPs with relatively high Fst values (> 0.7; S1 Fig). Most of the SNPs with high Fst values overlapped with those identified in the first approach [i.e., step 5(b) above], except for a few SNPs (N = 9) with low Fst values (meaning less informative SNPs), all of which were from *M*. *chevalieri*. Therefore, we excluded these SNPs (N = 9) and focused analysis on the ‘private SNPs’ or those considered as the most informative SNPs identified using the first approach (i.e., private SNPs). Besides, this species (i.e., *M*. *chevalieri*) is the most genetically divergent (see Fig 4), suggesting that a relatively few core SNPs are needed to distinguish from other *Macrobrachium* species. For the selected set of informative SNPs, we calculated allele frequency (MAF), observed, and expected heterozygosity for each population using the Hierfstat package [33] in R [34]. An overview of the SNP identification and validation is described in Fig 2.

### Validation of informative SNPs

We used three approaches to test whether selected ‘private SNPs’ are parsimonious or robust in discriminating *Macrobrachium* populations: a) principal component analysis (PCA) using PLINK software v1.9 [35], b) discriminant analysis of principal component (DAPC), and admixture analysis both (i.e., b and c) using the Adegenet package [36] in R [34]. Notably, PCA and DAPC methods are comparable except that the former aims to discern the overall variability in the population (i.e., within- and between-group variability), while the latter focuses on distinguishing between-group components [37]. Another difference between these methods is that PCA requires a priori definition of groups, whereas DAPC does not require this prior assumption of population clusters. In addition, unlike PCA, DAPC allows probabilistic assignment of individuals into their respective clusters [37]. However, both methods are similar in that they use a multivariate approach to cluster individuals, unlike Bayesian-clustering methods of, say the ADMIXTURE software [38]. Also, DAPC depends on the PCA approach as the first critical step in clustering [37]. Using the whole SNP set as a benchmark, we tested population assignment with two sets of informative SNPs:

- ‘private SNPs’ identified in step 5 (a) above (i.e., those identified in > 50 times of the repeated random subsets–‘private SNPs 50’).
- ‘private SNPs’ from step 5 (b) above (i.e., those detected in >80 of the repeated random subsets–‘private SNPs 80’). However, most of the ‘private SNPs Full’ panel overlapped with the ‘private SNPs 50’ panel (i.e., 178 out of 174 SNPs). Therefore, we only tested ‘private SNPs 50’ (N = 174 SNPs) and ‘private SNPs 80’ (N = 72 SNPs) against a benchmark marker set (N = 1,814 SNPs).

## Results and discussion

While the cost of genotyping has reduced considerably over the years, thanks to the rapid evolution of high-throughput technologies, it was not feasible to cost-effectively genotype a large population of highly diverse species such as *Macrobrachium* using dense genetic markers in our previous work (e.g., [19]). The objective of this study was to identify and test the effectiveness of a small set of SNPs for characterising *Macrobrachium* species. Consequently, we have demonstrated that it is possible to accurately discriminate between *Macrobrachium* species using a small suite of highly informative SNP panel (N = 72; see S1 Fig). Such cost-effective genotyping panels containing a small set of informative SNPs have been developed for smallholder farming systems in Africa (e.g., [39]).

We used several conventional statistics to choose a small set of highly informative SNPs for characterising *Macrobrachium* species: a) private SNPs [40]–defined as those segregating in only one population and fixed in others b) minor allele frequency, and c) SNPs with high Fst values > 0.70. We then validated prioritised SNPs using empirical (i.e., admixture analysis) and heuristic (PCA and DAPC) approaches. Overall, we found that the reduced set of 72 informative SNPs can classify *Macrobrachium* individuals into respective populations with 100% probability based on the ADMIXTURE results. Similarly, the PCA and DAPC methods showed good agreement when comparing clustering profiles of *Macrobrachium* species obtained from using a full set of SNPs (N = 1,814) versus a reduced set of informative SNPs (N = 72).

### Minor allele frequency (MAF)

**Fig 3:**
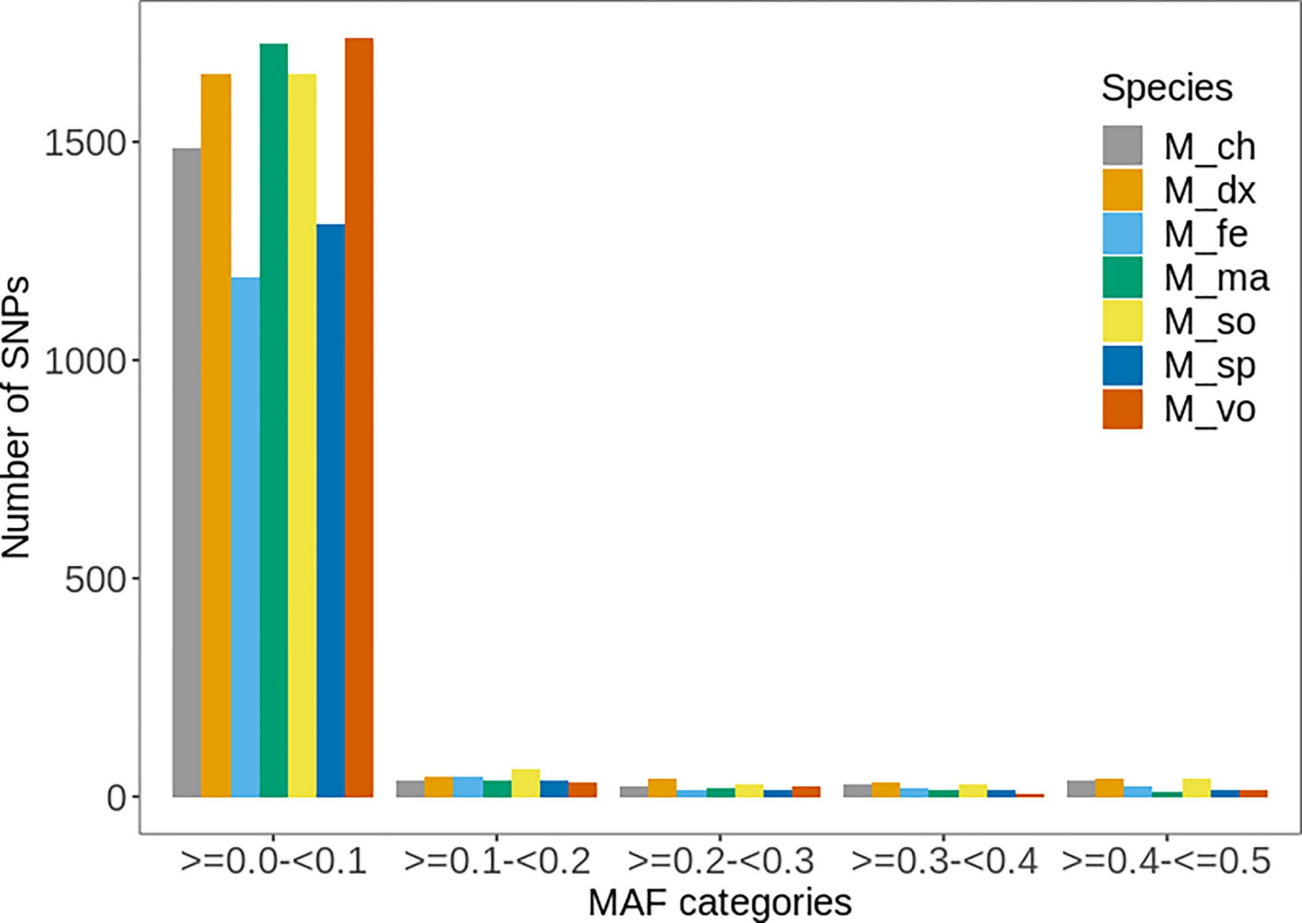
Distribution of minor allele frequency (MAF) for different *Macrobrachium* species: *M*. *dux*, *M*. *macrobrachion*, *M*. *sollaudii*, *M*. *vollenhovenii*, *M*. *chevalieri; M*. *felicinum*, and *M*. *sp*.

MAF is an important metric for evaluating the informativeness of genetic variants and has been used to develop custom SNPs arrays in cattle and other species (e.g., [41]). Fig 3 shows the distribution of MAF for *Macrobrachium* populations that were computed separately for each population. Most of the SNPs have low minor allele frequency (i.e., MAF < 0.1) across populations. However, a sizable number (N = 243) have relatively high MAF values (MAF > 0.1). These variants can be prioritised when designing the genotyping panels since they are likely to yield the greatest advantage in terms of distinguishing different *Macrobrachium* populations. Notably, the high proportion of SNPs with low MAF (i.e., < 0.1) was expected since the *Macrobrachium* genome is still poorly annotated. This is comparable to other studies in cattle (e.g., [42]) that reported a larger proportion of SNPs with low MAF for indicine breeds (less genetically described breed) compared to well-known Holstein breeds.

### Species-informative ‘private SNPs’

**Fig 4:**
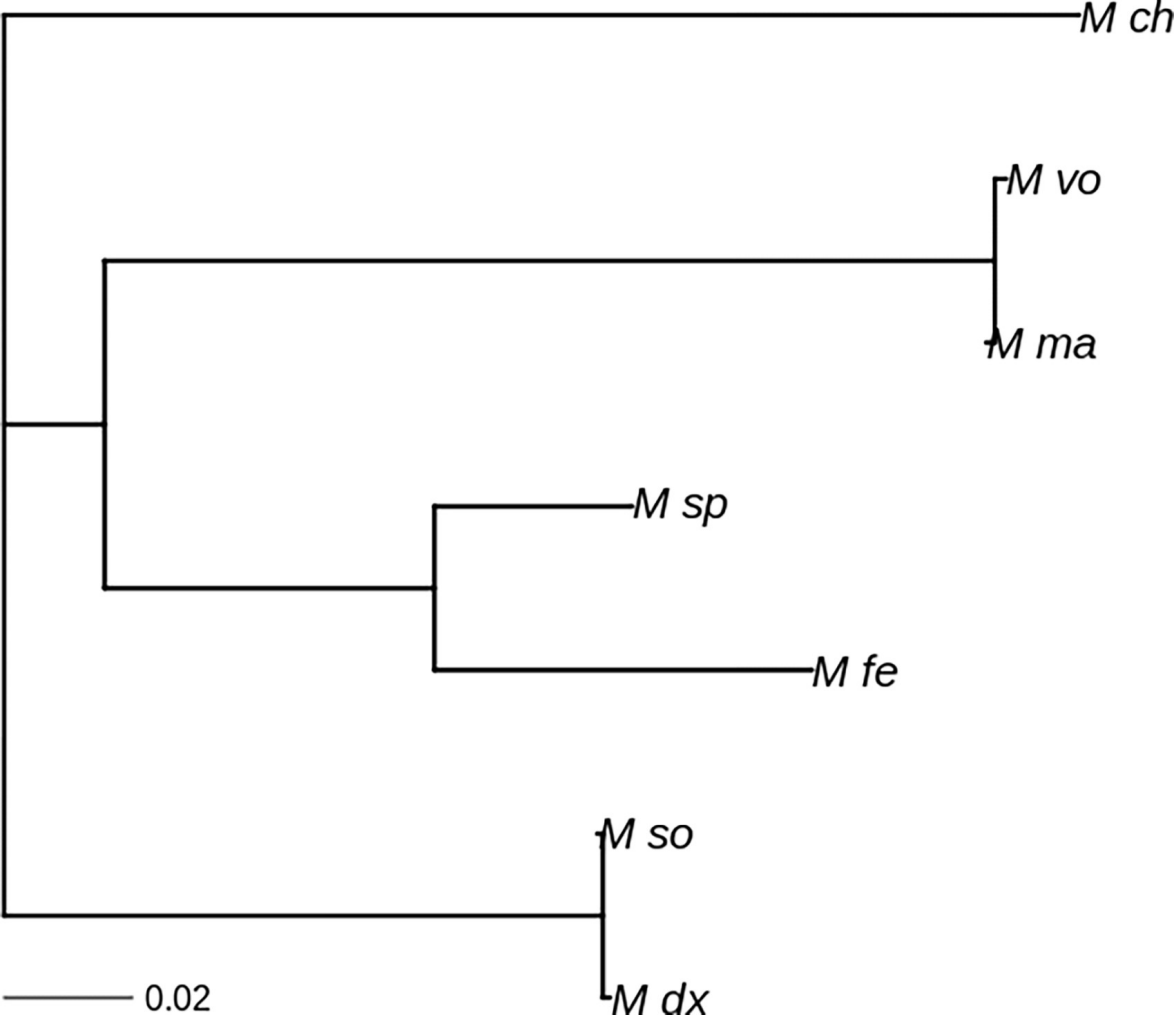
Phylogenetic tree obtained using 72 private SNPs for *Macrobrachium* species: *M*. *dux*, *M*. *macrobrachion*, *M*. *sollaudii*, *M*. *vollenhovenii*, *M*. *chevalieri; M*. *felicinum*, *and M*. *sp*. The phylogenetic tree for these *Macrobrachium* species using a larger set of SNPs (N = 1,814) is provided in [19].

Table 1 shows the summary statistics for the final set of ‘private SNPs’ (N = 72) identified in this study from a starting full set of 1,814 SNPs. Notably, these SNPs (i.e., N = 72) represent those identified from repeated re-sampling analysis (‘private SNPs 80’; see Methods) considered stable or of high-quality; therefore, more relevant for species population assignment. The number of ‘private SNPs’ ranged from 2 (*M*. *dux*) to 16 (*M*. *chevalieri*). The fact that we found only 2 ‘private SNPs’ for *M*. *dux* is not surprising considering that this species appears to be genetically closely related to *M*. *sollaudii* species based on phylogenetic analysis (Fig 4). The same case applies to *M*. *vollenhovenii* and *M*. *macrobrachion–*also genetically closely related species (Fig 4). While a possible reason for this close genetic relationship could be because of gene flow, our admixture results (Figs 7 and 8) suggest very limited admixture among these species. Alternatively, a more plausible reason could be that these species [i.e., *M*. *vollenhovenii* versus *M*. *macrobrachion* and *M*. *sollaudii* versus *M*. *dux*] are conspecific, meaning that classifying them as separate species using morphological keys could be misleading. We found most of the *M*. *sollaudii* samples were males, whereas *M*. *dux* were mainly females [see [19]]. Notably, the few *M*. *sollaudii* individuals classified as females were all young or juveniles. Overall, these observations suggest that it is highly likely that the morphological key is perhaps separating males and females of the same species.

**Table 1:** Summary statistics of the ’private SNPs’ (‘private SNPs80’; N = 72) identified in the study.

Population	N samples	‘private SNPs’*	MAF^⸸^	Obs (Het)	Exp (Het)	Fst values‡
*M*.* chevalieri*	13	16 (60)	0.05–0.48 (0.12)	0.073	0.076	0.75–0.91
*M*.* dux*	18	2 (23)	0.38–0.38 (0.38)	0.002	0.002	0.98, 0.96
*M*.* felicinum*	5	14 (24)	0.05–0.41 (0.19)	0.09	0.079	0.83–0.98
*M*.* macrobrachion*	17	13 (26)	0.05–0.47 (0.26)	0.01	0.016	0.91–0.98
*M*.* sollaudii*	18	8 (17)	0.05–0.38 (0.31)	0.008	0.009	0.85–0.98
*M*. *sp*	5	11 (16)	0.07–0.43 (0.26)	0.062	0.057	0.84–0.98
*M*.* vollenhovenii*	17	8 (7)	0.06–0.47 (0.27)	0.007	0.006	0.85–0.98

*The ‘private SNPs’ in brackets represents those identified from the less stringent repeated cut-off after repeat runs (i.e., ‘private SNPs50’; N = 174)

^**‡**^Fst values were computed based on Weir & Cockerham (1984) using PLINK software (Purcell et al., 2007); MAF–minor allele frequency

^⸸^Values in brackets represents average MAF.

Therefore, future studies with large sample sizes are needed to conclusively determine if these closely related individuals belong to the same species.

If indeed these species (i.e., *M*. *sollaudii* versus *M*. *dux*) are separate but with similar genetic relationship, then it means that many SNPs are required to discriminate between species. In contrast, we need a smaller number of informative SNPs to distinguish *M*. *chevalieri* versus other species, given that this species is genetically divergent compared to other species (Fig 4). This is consistent with the work of [43] in which the authors show that relatively more SNPs are required to characterise closely related cattle breeds. As such, we recommend further work with a larger sample size to identify more core SNPs, particularly for closely related *Macrobrachium* species identified in this study.

To date, the domestication and commercial aquaculture of *Macrobrachium* prawns have not been successful in Africa, unlike other species such as *M*. *rosenbergii*, which is widely cultured in other parts of the world [44]. However, work is currently underway in Cameroon to breed *M*. *vollenhovenii* as a food resource for humans (J. Makombu; personal communication) and as a biocontrol species for Schistosomiasis –a serious parasitic disease affecting humans ([45]). *M*. *vollenhovenii* species is often preferred for aquaculture because the adults are usually bigger compared to other *Macrobrachium* prawns. An attempt to crossbred *M*. *vollenhovenii* and *M*. *rosenbergii* by [46] was unsuccessful. Interestingly, in our field sampling, we found some *M*. *macrobrachion* adult individuals of the same size as *M*. *vollenhovenii* species. As noted earlier, we think that these two species are conspecific. This is supported by the phylogenetic tree (Fig 4) and the admixture results (Fig 7). While we identified 14 and 8 informative SNPs for *M*. *macrobrachion* and *M*. *vollenhovenii* species, respectively, it may be necessary to consider a smaller set of core SNPs for characterising these species, if it is conclusively established that they are indeed the same species of *Macrobrachium*.

The average MAF calculated from the ‘private SNPs’ (N = 72) [based on a combined dataset for all *Macrobrachium* species] ranged from 0.12 (*M*. *chevalieri*) to 0.38 (*M*. *dux*). Similarly, the observed and expected heterozygosity values were low, with the average estimates of 0.036 and 0.035. On the other hand, the Fst values were high (> 0.7) for this ‘private SNP’ set. The high Fst (> 0.7) and MAF (i.e., > 0.1) cut-off for ‘private SNPs’ chosen in this study suggests that they are highly informative for characterising *Macrobrachium* species.

Other studies have also identified core marker sets for characterising various species, including humans [21], cattle [23], wildlife [22], and plants [25]. For example [23], chose 48 and 96 informative SNPs for cattle from the 50k SNP chip based on the principal component analysis and machine learning methods (random forest). In recent work [24], followed a similar approach as [23] and identified a small set of informative SNPs for pigs from the porcine 60k array. While we discovered a total of 72 ‘private SNPs’ in this study, even a smaller number of high-quality SNPs is desirable to minimize genotyping costs for *Macrobrachium* species. However, a larger sample size is needed for use in prioritising more informative SNP set for routine genotyping *Macrobrachium* species.

### Validation of informative SNPs

#### PCA and DAPC using full set of SNPs

**Fig 5:**
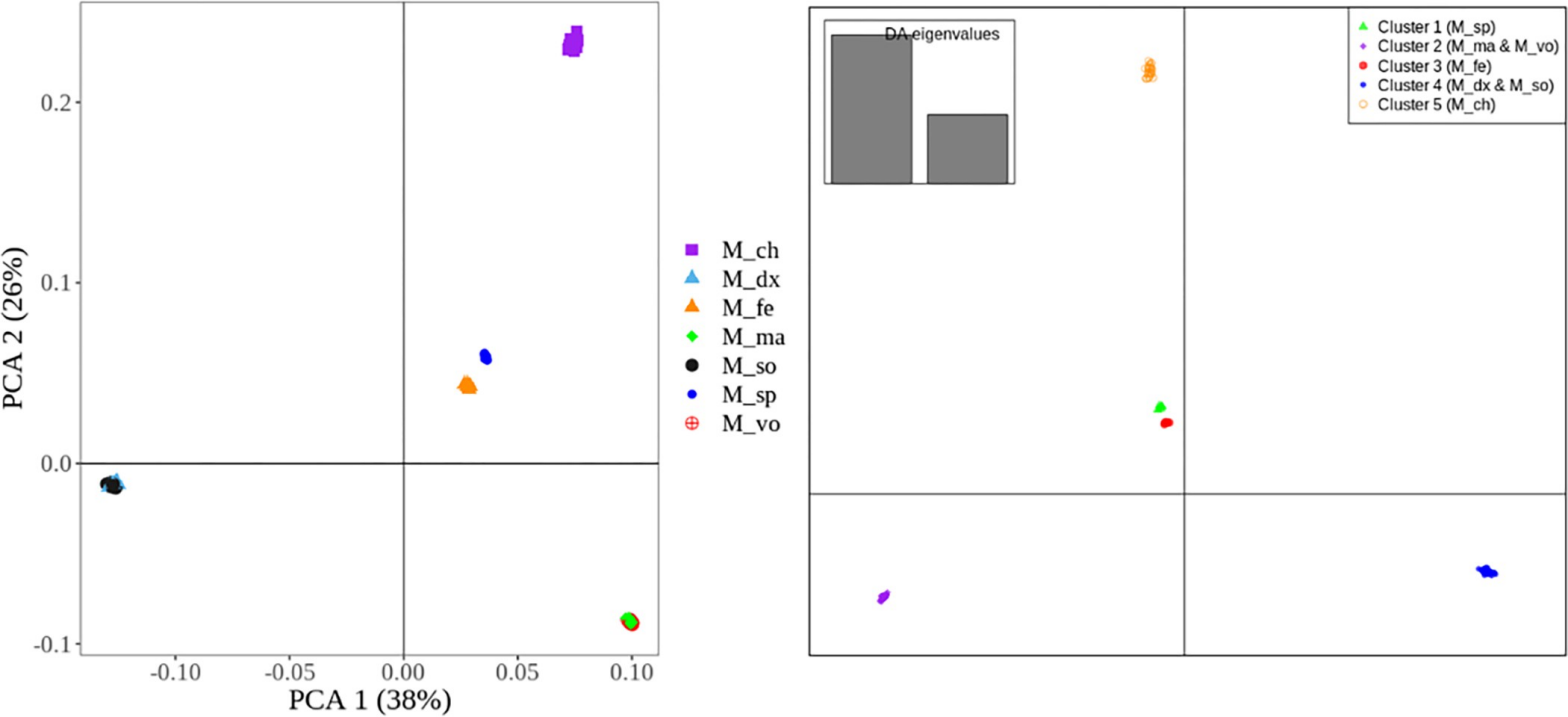
PCA (left plot) and DAPC (right plot) obtained from using a full set of SNPs (N = 1,814). PCA–principal component analysis; DAPC–discriminant analysis of principal components. *M_ch–M*. *chevalieri;M_dx–M*. *dux;M_fe–M*. *felicinum; M_ma–M*. *macrobrachion; M_so–M*. *sollaudii; M_sp–M*. *sp; M_vo–M*. *vollenhovenii*.

We used the results from the full set of SNPs for PCA and DAPC analysis as a benchmark to see how well different species are classified compared to the reduced set of core markers. Fig 5 shows the PCA and DAPC plots obtained when using a full set of SNPs (N = 1,814). The PCA plot in this study mirrors that reported by [19], in which five *Macrobrachium* populations were reported with the following clusters: *M*. *dux* and *M*. *sollaudii* (cluster 1); *M*. *macrobrachion* and *M*. *vollenhovenii* (cluster 2); *M*. *chevalieri* (cluster 3); *M*. *felicinum (cluster 4); M*. *sp* (cluster 5). This compares well with the results from the DAPC analyses when assuming 5 clusters of *Macrobrachium* species (Fig 5). In addition, these results are consistent with those from phylogenetic analysis discussed earlier (Fig 4). This phylogenetic profile is consistent with the one reported by [19] using a large set of 1,814 SNPs. Notably, these plots will be used as the basis to compare how well clustering performs when using the reduced set of ‘private SNPs’.

### PCA and DAPC using informative SNPs

**Fig 6:**
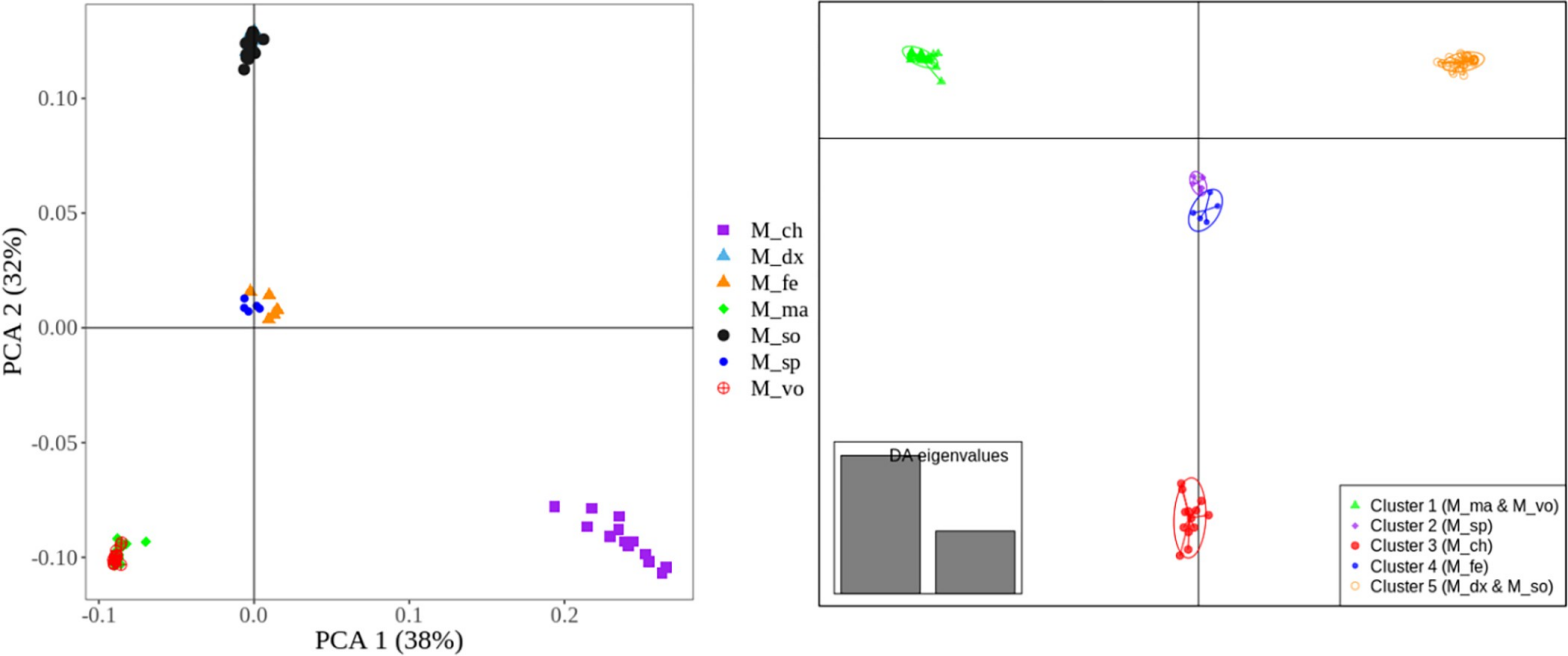
PCA (left plot) and DAPC (right plot) obtained from using the reduced set of ‘private SNPs’ (N = 72 SNPs, called ‘private SNPs80’ panel; see Methods). PCA–principal component analysis; DAPC–discriminant analysis of the principal components. *M_ch–M*. *chevalieri; M_dx–M*. *dux; M_fe–M*. *felicinum; M_ma–M*. *macrobrachion; M_so–M*. *sollaudii; M_sp–M*. *sp; M_vo–M*. *vollenhovenii*.

Fig 6 shows the PCA and DAPC plot obtained from the reduced set of ‘private SNPs’ (N = 72) considered as more stable or high-quality (i.e., the ‘private SNPs’ called ‘private SNPs 80’; see Methods). For PCA, these SNPs clearly distinguished four groups of *Macrobrachium* species with *M*. *felicinum* and *M*. *sp* appearing as one cluster, which contrast with the results obtained from the using full set of SNPs (as described above). However, the plot for PC1 versus PCA3 using ‘private SNPs 80’ clearly separated these species into two distinct populations (S1 Fig), indicating a total of five *Macrobrachium* species.

The above results from PCA (Fig 5), are consistent with those from the DAPC method (Fig 6) where *Macrobrachium* species were clustered into five populations. Notably, the individuals in Fig 5 above (i.e., benchmark results obtained from using the full set of SNPs, N = 1,814) appear as tightly clustered within their respective groups compared to those observed when using the ‘private SNPs’ in Fig 6. Overall, these results suggest that a small set of core SNPs can accurately separate *Macrobrachium* populations. Nonetheless, an obvious limitation of our study is the fact that the population used to discover informative SNPs and validation of this SNP set were the same. As such, future work using an independent sample is needed to confirm the discriminatory power of the selected core SNPs.

#### Admixture/membership classification using the full set of SNPs

**Fig 7:**
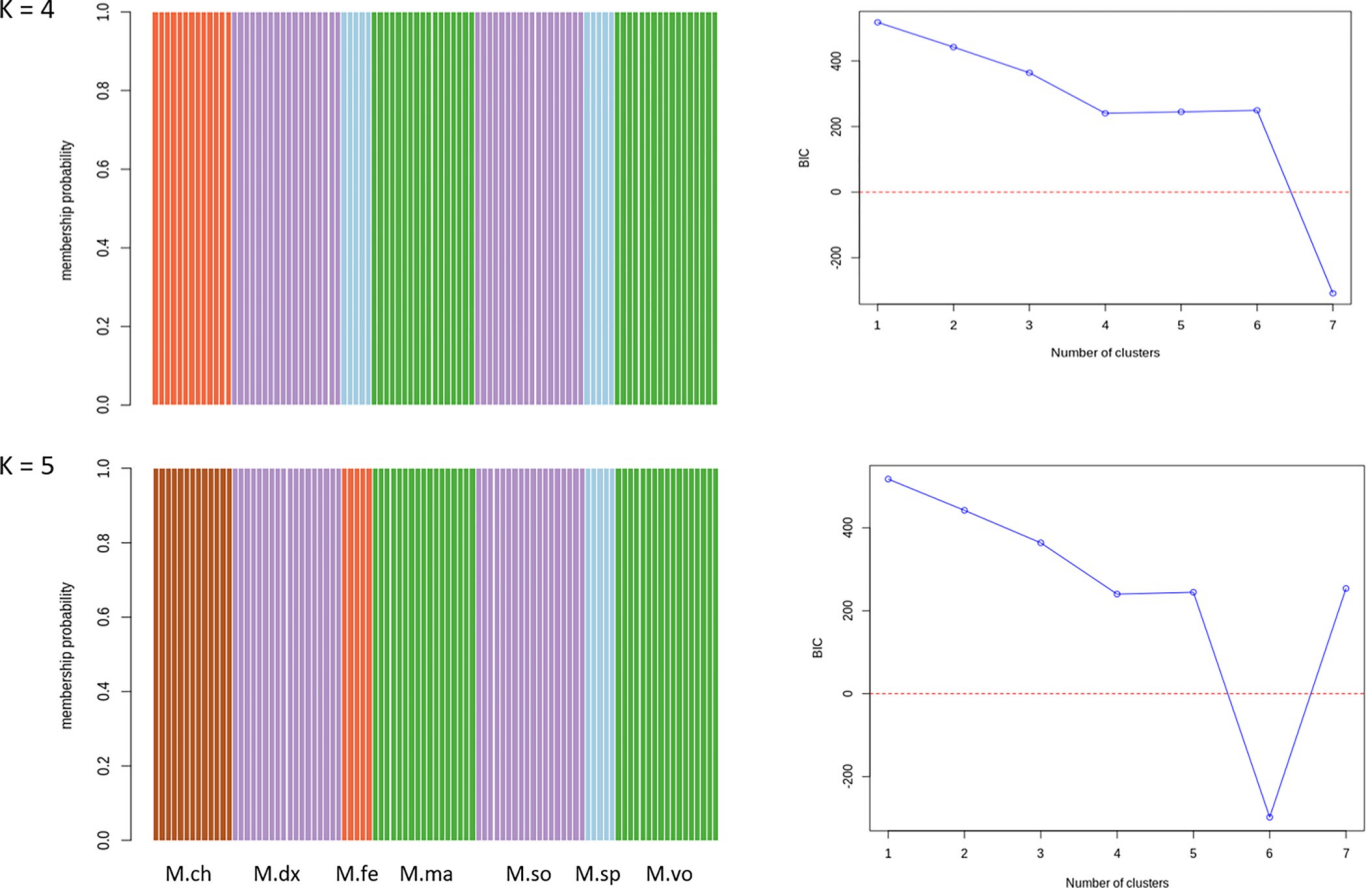
Cluster membership classification of *Macrobrachium* species and the Bayesian information criterion (BIC) plot obtained from the full set SNPs (N = 1,814) using the Adegenet package assuming four (K = 4) and five (K = 5) populations. Each bar in the admixture plot (left) represents an individual: M.ch–*M*. *chevalieri;* M.dx–*M*. *dux;* M.so*–M*. *sollaudii;* M.fe*–M*. *felicinum;* M.sp*–M*. *sp; M*.*ma–M*. *macrobrachion;* M.vo*–M*. *vollenhovenii*.

Apart from PCA and DAPC, we also performed admixture analysis to validate the prioritised set of SNPs. Fig 7 shows the admixture results obtained from using the full set of SNPs (N = 1,814), which we considered as the benchmark for subsequent analyses using the reduced set of ‘private SNPs’. The Bayesian information criterion (BIC) plot showed at least 4 to 6 populations of *Macrobrachium* species in the dataset based on the line of deflection in Fig 7. When assuming four groups (K = 4) as the optimal representation of the species in the dataset, we found that all the individuals clustered into their respective groups with 100% probability (Fig 7). This is comparable to the work of [19] when assuming the same K value (i.e., K = 4). These results also mimic those obtained from DAPC (assuming four clusters) analysis described earlier (Fig 5). By looking at Fig 5, *M*. *felicinum* and *M*. *sp* were separated into different groups when assuming K = 5, which somewhat differs from the results of [19], where these two species remained as one group at K = 5, most likely due to the different methods used for admixture analyses. Here, we used the Adegenet program by [36], while [19] used the ADMIXTURE program [38]. The Adegenet program uses discriminant analysis of principal components (DAPC) to infer population clusters, while the ADMIXTURE program applies the Bayesian clustering method. Another difference between the two programs is that the Adegenet uses the K-means algorithm and model selection to find the optimal number of clusters. In contrast, the ADMIXTURE program requires a *priori* definition of the best number of clusters in a dataset. The Adegenet program is designed to maximise between-group difference over within-group difference [37]. Regardless of the program used in the analysis, it is important to note, however, that the findings are comparable to, and are presented in, those of [19].

#### Admixture/membership classification using reduced set of ‘private SNPs’

**Fig 8:**
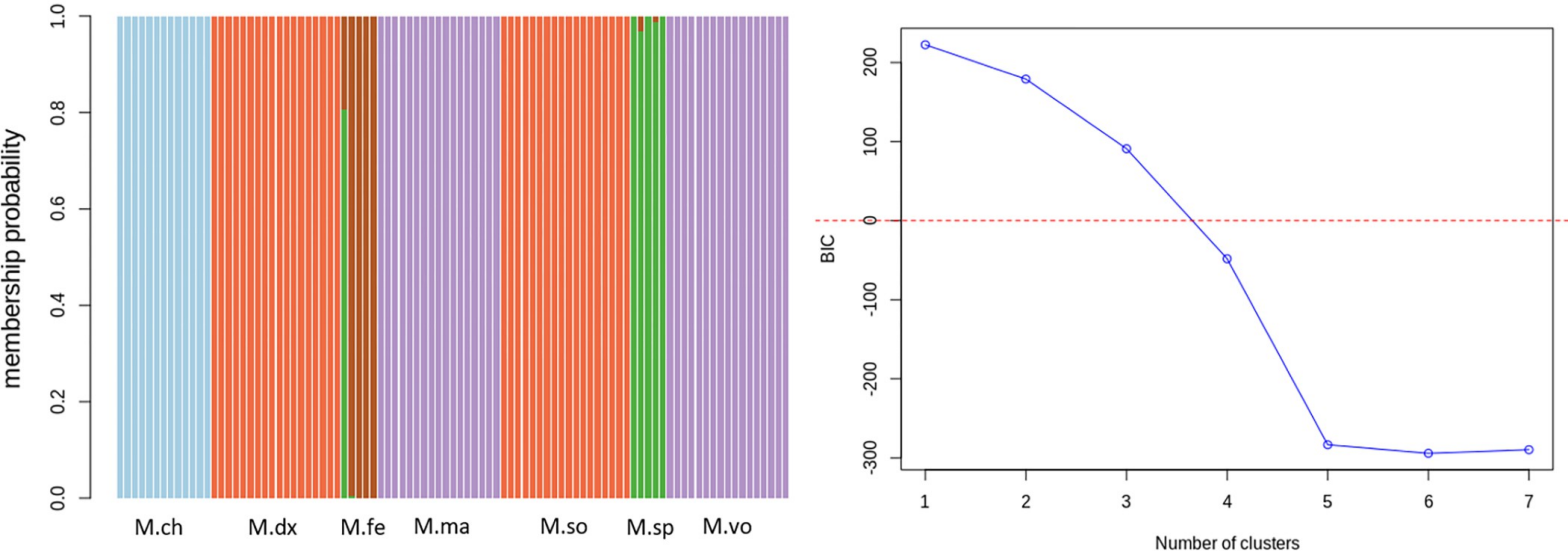
Cluster membership classification of *Macrobrachium* species and the Bayesian information criterion (BIC) plot obtained from a reduced set of informative or ‘private SNPs’ (N = 72 SNPs) using Adegenet package [36] assuming five clusters (K = 5). Each bar in the admixture plot (left) represents an individual: M.ch*–M*. *chevalieri;* M.dx*–M*. *dux;* M.so*–M*. *sollaudii;* M.fe*–M*. *felicinum;* M.sp*–M*. *sp;* M.ma*–M*. *macrobrachion;* M.vo*–M*. *vollenhovenii*.

Fig 8 shows the admixture results obtained from using a reduced set of ‘private SNPs’ (N = 72 SNPs) described in Table 1. The BIC plot clearly shows that assuming five populations is the most parsimonious to the dataset based on the point-of-line deflection. We, therefore, used K = 5 to display admixture proportions for each sample (Fig 8). By looking at the admixture results, most individuals were classified into their distinct groups (N = 5) with 100% probability, except for a few admixed individuals within *M*. *felicinum* and *M*. *sp* populations. These results are consistent with those found when using the full set of SNPs (N = 1,814) described above (Fig 5) and those from the PCA and DAPC analyses (Fig 4). The ADMIXTURE results obtained from the reduced set of 72 core SNPs were also comparable with those from another set of ‘private SNPs’ panel (N = 174; see Methods for description) (S2 Fig).

For comparison, we also assessed ancestry classification using the ADMIXTURE software [38] for the reduced set of ‘private SNPs’ (N = 72). Consequently, we found consistent results with those from the Adegenet package [36] [which is based on the discriminant analysis], when assuming K = 5 with the ADMIXTURE software. As seen in the S3 Fig (i.e., results from the ADMIXTURE software), the *Macrobrachium* species were clustered into five groups (at K = 5) with almost 100% ancestry probability in each group.

As discussed earlier, we have used conventional methods (MAF, Fst, and allele frequency differentials) to select informative SNPs for *Macrobrachium* species. Alternatively, more advanced methods such as machine learning (e.g., [23]) could be used to identify core SNPs for *Macrobrachium* species. However, the fact that the genome for *Macrobrachium* is still poorly annotated makes it difficult to apply such methods. For example, in this study most individuals had missing genotypes in one or more SNPs. Similarly, more recent supervised methods (e.g., [30]) rely on linkage disequilibrium (LD), which is not possible to apply for our study species with poorly annotated genomic map. Notably, most (82%; N = 1,489) of our SNPs lacked chromosomal positions. Moreover, of the 72 informative SNPs, only 20 SNPs were mapped to their chromosomal location (see S1 Table) based on the recent genome assembly of *M*. *nipponense* [47]. With the availability of the comprehensive genome annotation, future work could leverage LD or pedigree information to prioritise informative marker set for *Macrobrachium* species.

We used group re-sampling approach to select high-quality or stable set of SNPs for characterising *Macrobrachium* species and to guard against false positives (see Methods). However, the small sample sizes within each *Macrobrachium* species makes such re-sampling efforts less effective, meaning a large sample set is needed to identify new informative SNPs and confirm our results. In addition, methods which combine group resampling and machine learning approaches (e.g., [48]) could be tested in future studies for *Macrobrachium* species. A new validation dataset will have to be provided to test these approaches and the discriminating power of the selected SNP panels.

The core SNPs chosen in this study could be extremely useful in the genetic characterisation of cryptic species of *Macrobrachium*. For example, by using BLAST tool [32], we found that some of the allele sequence for the 72 informative SNPs (see S1 Table) mapped to the protein spaetzle-like gene. This gene was found to play a role in the development of dorsal-ventral pattern of the drosophila melanogaster embryos [49]. Also, some of the allele sequences mapped to the CD209 gene associated with immune response and stress in prawn species [50, 51]. In our previous work [19], we identified a potentially new species of *Macrobrachium*, which we named *M*. *sp* (Fig 4). This species is morphologically closely related to another species of *Macrobrachium* called *M*. *felicinum* (see the images in Fig 1). Considering that *M*. *sp* was identified from only a few genotyped samples (N < 20), with large sample sizes availed, it is highly likely that new species are yet to be correctly described. As such, the core SNPs from this study could facilitate the cost-effective screening of thousands of *Macrobrachium* individuals to identify new species for breeding purposes. In addition, given the current scenario of climate changes, the findings of this study can facilitate documenting new *Macrobrachium* species that are potentially at risk of extinction to inform conservation efforts before they are lost.

## Conclusion

Overall, the results in this study show that we can use a small set of 72 highly informative SNPs to characterise *Macrobrachium* species from the coastal area of Cameroon with 100% accuracy. This marker set could facilitate the genetic characterisation of *Macrobrachium* species in a cost-effective way for conservation and breeding purposes. However, further work is needed to validate the core SNPs identified in this study. A large sample size will have to be collected to facilitate such validation.

## Supporting information

S1 Table Informative SNP markers (N = 72) for characterising Macrobrachium species. (XLS)

S1 Fig PCA plot obtained from a full set of 1,814 SNPs (A), 174 private SNPs (B) and 72 ‘private SNPs 80’ (C). M_ch–M. chevalieri;M_dx–M. dux;M_fe–M. felicinum; M_ma–M. macrobrachion; M_so–M. sollaudii; M_sp–M. sp; M_vo–M. vollenhovenii.
(TIF)

S2 Fig Admixture results obtained from using 174 ’private SNPs’ based on the ADMIXTURE software. (TIF)

S3 Fig Admixture results obtained from using 72 ’private SNPs’ based on the ADMIXTURE software. (TIF)
