# Supplementary figures and images for: Species-informative SNP markers for characterising freshwater prawns of genus Macrobrachium in Cameroon
Source: PLoS One. 2022 Oct 3;17(10):e0263540. doi: 10.1371/journal.pone.0263540 (PMC9529149; doi:10.1371/journal.pone.0263540)

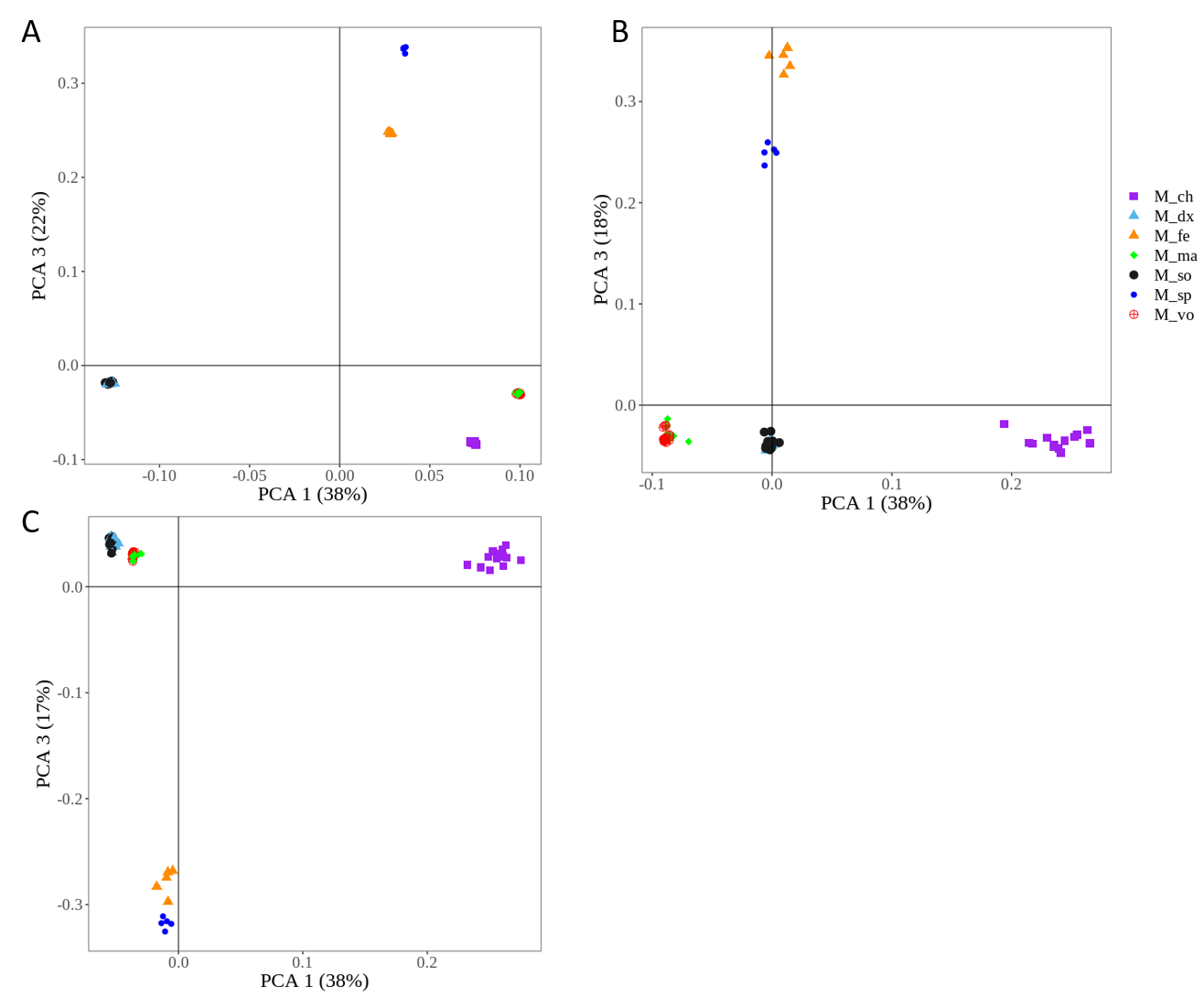

Supplement: S1 Fig — PCA plot obtained from a full set of 1,814 SNPs (A), 174 private SNPs (B) and 72 ‘private SNPs 80’ (C). M_ch–M. chevalieri;M_dx–M. dux;M_fe–M. felicinum; M_ma–M. macrobrachion; M_so–M. sollaudii; M_sp–M. sp; M_vo–M. vollenhovenii. (TIF) [file pone.0263540.s002.tif]

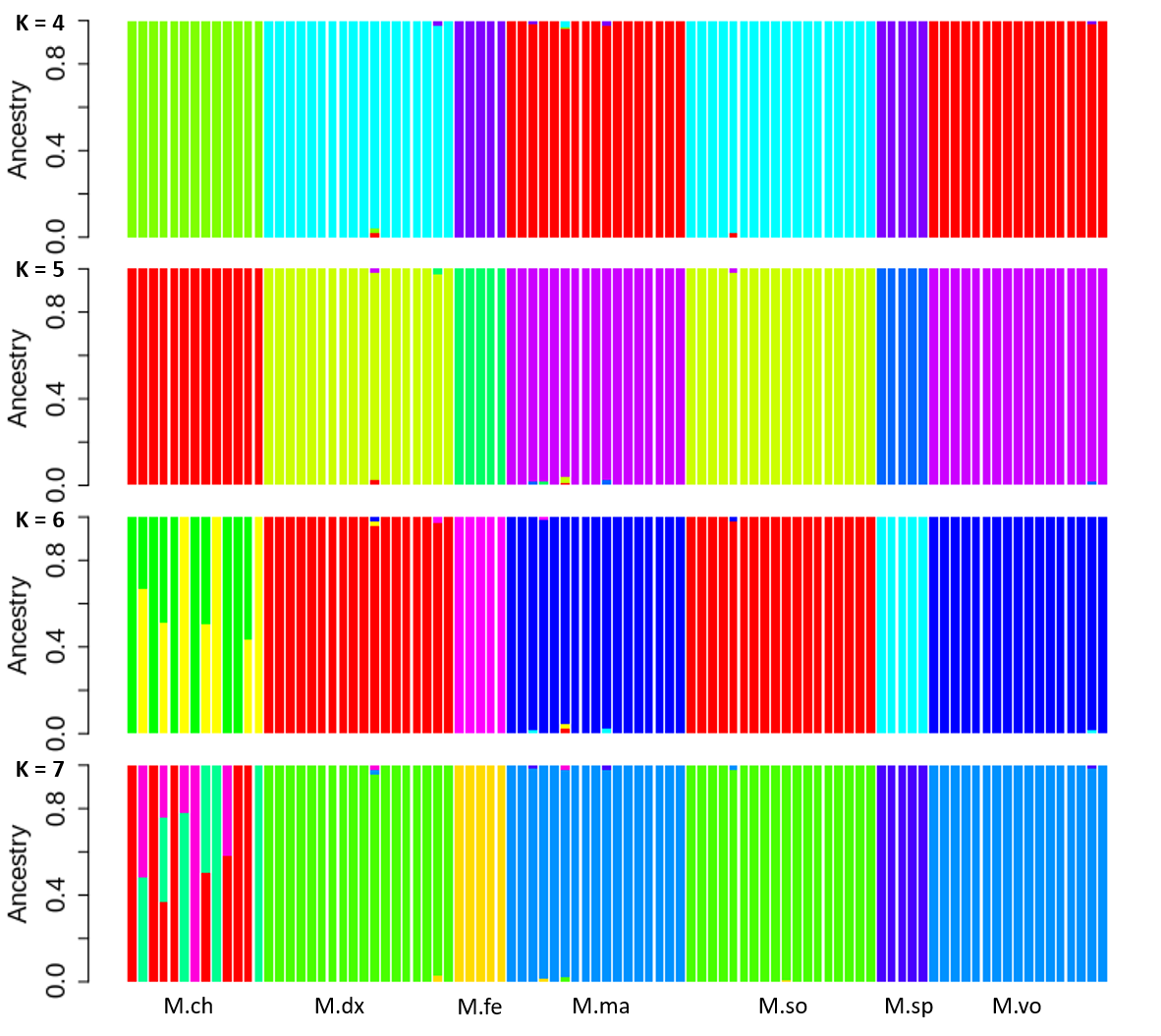

Supplement: S2 Fig — (TIF) [file pone.0263540.s003.tif]

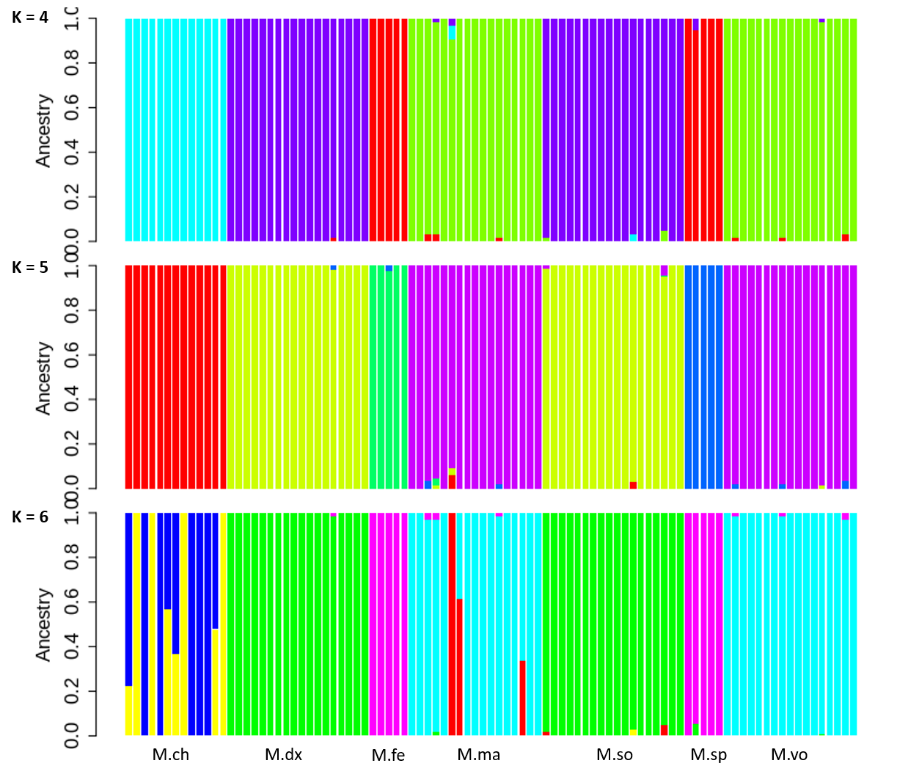

Supplement: S3 Fig — (TIF) [file pone.0263540.s004.tif]
